# Non-interventional Feasibility Assessment for Fast-Track Cardiac Anesthesia

**DOI:** 10.7759/cureus.34392

**Published:** 2023-01-30

**Authors:** Hülya Yiğit, Zeliha A Demir, Eda Balcı, Levent H Mavioğlu

**Affiliations:** 1 Anesthesiology, Ankara City Hospital, Ankara, TUR; 2 Cardiac/Thoracic/Vascular Surgery, Ankara City Hospital, Ankara, TUR

**Keywords:** early recovery, icu, pandemic, cardiac surgery, fast-track cardiac anesthesia

## Abstract

Background

The introduction of fast-track extubation procedures following cardiac surgery has significantly shortened hospitalization duration in intensive care units (ICUs). Early extubation is the most crucial step in getting out of the ICU early and providing ideal patient circulation. In times of crisis such as pandemics, it is vital to provide rapid flow through the hospital to prevent the postponement or inability to operate on patients awaiting surgery. This study aimed to determine the obstacles to early extubation in patients undergoing cardiac surgery and the perioperative characteristics that were affected in terms of fast-track extubation.

Methodology

This was an observational, cross-sectional study with data collected prospectively from October 1 to November 30, 2021. Preoperative data and comorbidities were recorded. Intraoperative and postoperative data were recorded and analyzed. Intraoperative cross-clamp duration, cardiopulmonary bypass duration, length of operation, and erythrocytes (red blood cells) transfused were recorded for each patient. Early postoperative clinical conditions were defined in patients whose mechanical ventilation duration exceeded eight hours (such as pulmonary complications, cardiovascular complications, renal complications, neurological conditions, and infective complications ). The length of ICU stay (hours), length of hospital stay (days), return to the ICU, reasons for return to the ICU, and overall hospital mortality were investigated. A total of 226 patients were included in the study. Patients were divided into two groups: extubated within eight hours (FTCA, fast-track cardiac anesthesia) and late extubation (after eight hours) postoperatively, and the data were evaluated accordingly.

Results

While 138 (61.1%) of the patients were extubated in eight hours or less, 88 (38.9%) patients were extubated after more than eight hours. The most common complications (55.7%) in patients with late extubation were cardiovascular complications, followed by respiratory complications (15.9%), and the surgeon’s refusal (15.9%). In the logistic model created with the independent variables affecting the extubation time, the American Society of Anesthesiologists score and red blood cell transfusion were risk factors for longer extubation time.

Conclusions

In our research to reveal the feasibility of and barriers to FTCA, it was found that cardiac and respiratory problems were the most common reasons for delayed extubation. Due to the refusal of the surgical team, it was observed that some patients remained intubated despite meeting the FTCA requirements. It was considered the most improvable obstacle. Regarding cardiovascular complications, the team should aim to optimally control patient comorbidities in the preoperative period, reduce the use of red blood cell transfusions, and ensure that the entire team is updated on current extubation protocols, in particular surgeons and anesthesiologists.

## Introduction

The goal of fast-track cardiac anesthesia (FTCA) is to have the patient extubated within cardiac anesthesia. In FTCA, the patient is extubated within six to eight hours after cardiac surgery and safely removed from the intensive care unit (ICU) within 24 hours. FTCA provides early tracheal extubation, shortens ICU and hospital stays, and reduces cost and postoperative complications [1]. The coronavirus disease 2019 (COVID-19) pandemic has resulted in worldwide staffing and ICU bed shortages. In addition, cardiac ICU staff has been redeployed to other sections of the hospital to cover shortages. As cardiac surgeries are often emergent, there are frequent ICU bed and staff shortages at the time of surgery. The implementation of FTCA can not only reduce costs and length of stay it can also help optimize scarce nursing and ICU bed resources. In the postoperative period, if the patients’ status is appropriate, they can be extubated within eight hours in FTCA programs [2]. Some patients fail to meet extubation criteria due to hemodynamics, cardiac, and respiration problems. For these reasons, a prospective, cross-sectional study was planned to focus on our standard daily practices and improve areas of failure.

This study aimed to examine the preoperative, intraoperative, and postoperative characteristics of patients who underwent cardiac surgery and investigate the causative factors in patients who could not be extubated within eight hours after surgery

## Materials and methods

This was an observational, cross-sectional study with prospectively collected data. The study was conducted following the principles of the Declaration of Helsinki. The study was approved by the Ethics Committee for Clinical Research at Ankara City Hospital (E1-22-2421, 23.02.2022). We included consecutive patients aged >18 years who underwent elective or emergency cardiac surgery from October 1 to November 30, 2021. Patients undergoing congenital cardiac surgeries were excluded from the study. Age, gender, body mass index, ejection fraction, and American Society of Anesthesiologists (ASA) physical status classification scores and European System for Cardiac Operative Risk Evaluation (Euro score II) were obtained. The following preoperative comorbidities were recorded: diabetes mellitus (DM), heart failure, hypertension, hyperlipidemia, hypercholesterolemia, chronic obstructive pulmonary disease (COPD), neurological disease, and chronic kidney failure.

Intraoperative cross-clamp duration, cardiopulmonary bypass (CPB) duration, length of operation, and erythrocytes (red blood cell, RBC) transfused were recorded for each patient. Surgical procedures were classified as follows: coronary artery bypass graft (CABG) surgeries, valve operations (involving one or more valves), aortic aneurysm repair and dissection surgeries, heart failure surgeries, atrial septal defect repair, and intracardiac myxoma surgeries.

Early postoperative clinical conditions were defined in patients whose mechanical ventilation duration exceeded eight hours. Pulmonary complications were defined as pneumonia, respiratory insufficiency, readmission, and tracheostomy. The diagnosis of pneumonia was made by examining the bronchogram in the areas where air density increased in the radiological examination. Respiratory failure was evaluated according to the presence of hypoxemia, hypercarbia in arterial blood gas, and the presence of conditions such as infection, fever, sepsis, respiratory drive mechanism, adequate hemoglobin level, cardiovascular stability, and electrolyte and metabolic balance. Readmission was defined as the retransmission of patients to the ICU. The tracheostomy requirement was evaluated in patients who could not be weaned from mechanical ventilation. Postoperative cardiovascular complications were defined as myocardial infarction (MI), failure, arrhythmia, ST-segment elevation, or non-ST-segment elevation (12-lead ECG performed every eight hours while in the ICU). Postoperative heart failure was defined as an inability to wean from CPB secondary to cardiac failure, pump failure implying CPB machine failure, and hemodynamic deterioration requiring active treatment with vasopressors/inotropes other than correction of volume or vascular resistance after weaning from CPB. Arrhythmia was defined as any arrhythmia requiring cardioversion or antiarrhythmic medication or disturbing hemodynamic stability. Renal complications were defined as acute renal complications requiring dialysis. Neurological conditions were defined as postoperative stroke-causing permanent or temporary neurological sequelae. Infective complications were defined as septicemia requiring antibiotherapy because of positive culture or sternal infection, leg infection, and urinary tract infection. The length of ICU stay (hours), length of hospital stay (days), return to the ICU, reasons for return to the ICU, and overall hospital mortality were investigated. In our center, patient blood management (PBM) has been practiced for a long time, and intravenous (IV) iron therapy is the established practice for patients with preoperative iron deficiency. PBM applications such as cell protection and tranexamic acid use are also performed in critical surgery patients, and intraoperative 7.5 g/dL hemoglobin value and postoperative 8.5 g/dL hemoglobin value are used as RBC transfusion thresholds.

The patients were divided into two groups, namely, those who met the FTCA criteria and those who did not. The data were evaluated accordingly.

Statistical analysis

Mean, standard deviation, median, minimum, and maximum values were calculated for continuous data, and percentage values were presented for discrete data. The Shapiro-Wilk test was used to examine the conformity of continuous data to normal distribution. To compare continuous data in two groups (extubation time more than eight hours and less than or equal to eight hours), the t-test was used for normally distributed data, and the Mann-Whitney U test was used for data that did not fit in a normal distribution. Chi-square and Fisher’s exact tests were used for group comparisons (cross tables) of nominal variables. The factors affecting the extubation time to be more than eight hours were analyzed using multivariate logistic regression (p < 0.01). SPSS Statistics version 20 (IBM Corp., Armonk, NY, USA) was used in the evaluations, and p < 0.05 was accepted as the statistical significance limit.

## Results

In the cross-sectional study of two consecutive months, 226 adult patients were included. Preoperative characteristics, comorbidities, type of operation, and intraoperative and postoperative data of the general patient population are presented in Table 1. Extubation time in 38.9% of the patients who did not meet the FTCA and 61.1% of patients who met the FTCA. The most common complications in patients who met and did not meet FTCA were cardiovascular system complications (55.7%), followed by respiratory system complications and surgeon complications both with rates of 15.9%. ICU return occurred in 7.1% of the patients. Respiratory system complications were the reason for 43.8% of ICU returns. Mortality was detected in 5.3% of the patients. The comparison of patients who met and did not meet the FTCA criteria is presented in Table 2 and Table 3. The mean extubation time was 7.04 ± 1.33 hours in patients who met the FTCA and 42.82 ± 86.39 hours in patients who did not meet the FTCA criteria. Valve surgery, aortic dissection, ascending aortic aneurysm, and left ventricular assist device/biventricular assist device (LVAD/BIVAD) surgery were more common in patients who did not meet the FTCA criteria (Table 3). Additionally, there were differences between the groups in terms of Euro Score, ejection fraction, duration of cross-clamp and CPB, duration of surgery, RBC transfused, length of ICU stay, length of hospital stay, return to the ICU, and mortality. ASA and RBC transfused were significantly higher in the group that did not meet the FTCA criteria (p = 0.002, p = 0.004, respectively).

**Table 1:** Patient characteristics. COPD = chronic obstructive pulmonary disease; CVD = cerebrovascular disease; CABG = coronary artery bypass graft; MVR/AVR = mitral valve replacement/aortic valve replacement; LVAD/BIVAD = left ventricular assist device/biventricular assist device; ASD = atrial septal defect; ICU = intensive care unit; COVID-19 = coronavirus disease 2019

Variables	Mean ± SD, Median (minimum-maximum)
Age, years	57.29 ± 13.12, 60 (15-81)
Body mass index, kg/m²	27.37 ± 3.65, 27.57 (16.79-38)
Euro Score II	0.97 ± 0.57, 0.78 (0.05-3.74)
Ejection fraction, %	52.41 ± 10.17, 55 (15-65)
Male gender, n (%)	159 (70.4)
ASA, n (%)	
1	3 (1.3)
2	131 (58)
3	85 (37.6)
4	7 (3.1)
Complications, n (%)	
Diabetes mellitus	61 (27)
Hypertension	155 (68.6)
Hyperlipidemia	44 (19.5)
COPD	13 (5.8)
CVD	2 (0.9)
Renal disease	8 (3.5)
Type of surgery, n (%)	
CABG	139 (61.5)
MVR/AVR	50 (22.1)
Aortic dissection	6 (2.7)
Ascending aortic aneurysm	13 (5.8)
LVAD/BIVAD	6 (2.7)
ASD/Myxoma	12 (5.3)
RBC transfused	92 (40.7)
Cross-clamp duration, minutes	81.82 ± 41.78, 74.5 (2-232)
Cardiopulmonary bypass duration, minutes	120.58 ± 52.24, 110 (28-426)
Operation duration, minutes	312.94 ± 61.57, 300 (180-580)
Time to extubation time, hour	20.97 ± 56.50, 8 (1-408)
Extubation time	
≤8 hour	138 (61.1)
˃8 hour	88 (38.9
Reasons for prolonged extubation (n = 88), n (%)	
Respiratory system complications	14 (15.9)
Cardiovascular system complications	49 (55.7)
Renal system complications	7 (8.0)
Neurological complications	3 (3.4)
Surgeon request	14 (15.9)
Infections	1 (1.1)
Length of stay in the ICU, hour	74.88 ± 140.4, 24 (24-1,152)
Length of stay in the hospital, day	8.84 ± 8.20, 6 (1-71)
Readmission to the ICU	16 (7.1)
Reasons for readmission to the ICU (n = 16), n (%)	
Respiratory system complications	7 (43.8)
Cardiovascular system complications	4 (25)
Renal system complications	1 (6.3)
Infections	3 (18.8)
COVID-19 disease	1 (6.3)
Mortality	12 (5.3)

**Table 2:** Comparison of patients with extubation time of eight hours or less and those with extubation time of more than eight hours. ASA = American Society of Anesthesiologists; COPD = chronic obstructive pulmonary disease; CVD = cerebrovascular disease; CABG = coronary artery bypass graft; MVR/AVR = mitral valve replacement/aortic valve replacement; ICU = intensive care unit

Mean ± SD or n (%)	Extubation ≤8 hours (n = 138)	Extubation >8 hours (n = 88)	P-value
Age, years	56.61 ± 13.54	58.35 ± 12.43	0.367
Body mass index, kg/m²	27.47 ± 3.32	1.17 ± 0.86	0.630
Euro Score II	0.88 ± 0.47	1.10 ± 0.68	0.002
Ejection fraction (%)	54.88 ± 7.28	48.55 ± 12.62	<0.001
ASA, Median (minimum-maximum)	2 (1-3)	3 (2-4)	<0.001
Male gender, n (%)	98 (71)	61 (69.3)	0.785
Diabetes mellitus, n (%)	32 (23.2)	29 (33)	0.107
Hypertension, n (%)	95 (68.8)	60 (68.2)	0.917
Hyperlipidemia, n (%)	31 (22.5)	13 (14.8)	0.154
COPD, n (%)	6 (4.3)	7 (8)	0.256
CVD, n (%)	1 (0.7)	1 (1.1)	1.000
Renal disease, n (%)	3 (2.2)	5 (5.7)	0.267
Type of surgery, n (%)			
CABG, n (%)	92 (66.7)	47 (53.4)	0.107
MVR/AVR, n (%)	28 (20.3)	22 (25)	
Others, n (%)	18 (13)	19 (21.6)	
Cross-clamp duration, minute	76.08 ± 35.29	90.82 ± 48.18	0.013
Cardiopulmonary bypass duration, minute	109.92 ± 42.34	137.30 ± 61.41	<0.001
Duration of surgery, minute	301.85 ± 53.83	330.34 ± 68.85	<0.001
Transfusion, n	38 (27.5)	54 (61.4)	<0.001
Length of stay in the ICU, hour	38.26 ± 72.12	132.96 ± 193.69	<0.001
Length of stay in the hospital, day	6.96 ± 4.35	11.83 ± 11.41	<0.001
Readmission to the ICU, n (%)	6 (4.3)	10 (11.5)	0.042
Mortality, n (%)	2 (1.4)	10 (11.4)	0.002

**Table 3:** Distribution of surgery type in patients with extubation time of eight hours or less and patients with more than eight hours of extubation. CABG = coronary artery bypass graft; MVR/AVR = mitral valve replacement/aortic valve replacement; LVAD/BIVAD = left ventricular assist device/biventricular assist device; ASD = atrial septal defect

Variable, N (%)	Extubation ≤8 hours (n = 138)	Extubation >8 hours (n = 88)
Type of surgery		
CABG, n (%)	92 (66.7)	47 (53.4)
MVR/AVR, n (%)	28 (20.3)	22 (25)
Aortic dissection, n (%)	2 (1.4)	4 (4.5)
Ascending aortic aneurysm, n (%)	5 (3.6)	8 (9.1)
LVAD/BIVAD, n (%)	0 (0)	6 (6.8)
ASD/Myxoma, n (%)	11 (8)	1 (1.1)

In the analysis of the risk factors affecting the group that did not meet FTCA criteria, the independent variables (DM, hyperlipidemia, ASA score, euro score, ejection fraction, type of surgery, cross clamp-CPB duration, surgical time, the number of transfused patients) were included in the multivariate logistic regression analysis to obtain the final model (Table 4). After analysis, ASA score and RBC transfusion were risk factors for failure to achieve FTCA. RBC transfusion increased the risk of failure to meet the FTCA criteria by 2.745 times (p < 0.01).

**Table 4:** Logistic regression model for risk factors affecting extubation time of more than eight hours. DM = diabetes mellitus; ASA = American Society of Anesthesiologists; EF = ejection fraction; RBC = red blood cell; SE = standard error; CI = confidence interval

Variable, N	Regression coefficient (SE)	OR	95% CI		P-value
DM	0.283(0.396)	1.327	0.610	2.883	0.476
Hyperlipidemia	0.517 (0.456)	1.677	0.686	4.101	0.205
ASA	1.178 (0.386)	3.247	1.524	6.918	0.002
Euro Score	-0.289 (0.317)	1.335	0.717	2.487	0.362
EF, %	-0.028 (0.021)	1.028	0.986	1.071	0.191
Valve surgery	0.416 (0.460)	1.517	0.616	3.735	0.365
Other types of surgery	0.440 (0.527)	1.552	0.552	4.362	0.404
Cross-clamp duration, minute	-0.009 (0.010)	1.009	0.990	1.029	0.347
CPB bypass duration, minute	0.012 (0.009)	1.012	0.995	1.030	0.156
Duration of surgery, minute	0.003 (0.004)	1.003	0.995	1.011	0.466
RBC transfused	1.010 (0.351)	2.745	1.379	5.466	0.004

## Discussion

This study aimed to determine the obstacles to early extubation in patients undergoing cardiac surgery and investigate the perioperative characteristics in terms of FTCA. ASA score and RBC transfusion were significant predictors of the failure of this protocol.

During the pandemic, the number of postoperative ICU beds for cardiac surgery has been reduced, as well as the number of staff working, as many of them have been diverted to combat the pandemic. Rapid recovery of patients after cardiac surgery is becoming more and more important for both shortening the waiting time for surgery (for example, during the pandemic) and reducing costs [3,4]. FTCA offers a higher-quality alternative to standard cardiac postoperative care and provides better efficiency, without sacrificing safety or optimal clinical outcomes [5,6].

Evidence from an extensive review of the FTCA literature shows no difference in adverse events compared to the standard approach [4,7,8]. For many years, various cardiovascular surgery teams in our hospital have preferred FTCA patients who meet the appropriate conditions. In parallel, approaches have been introduced in our anesthesia clinic to use more short-acting analgesic and anesthetic agents, use regional analgesia techniques more frequently, and avoid other drugs with long-acting and cumulative effects. For example, we avoided midazolam due to the possibility of postoperative delirium and fentanyl due to its prolonged and cumulative effect. We have been following the anesthesia protocol under the guidance of BIS with a remifentanil-propofol infusion or remifentanil-sevoflurane. In addition to general anesthetic techniques, we employ various regional techniques such as erector spinae plane blocks, serratus anterior plane blocks, and IV paracetamol for postoperative pain relief.

The accumulation of patients waiting for surgery caused chaos, as elective surgeries were globally stopped during the pandemic, and doctors and nurses shifted to areas of combating the pandemic. The importance of using the operating room and ICUs with maximum efficiency was again emphasized during the pandemic. Therefore, the expected benefit of this study is to examine the perioperative characteristics of patients undergoing cardiac surgery and make targeted improvements if modifiable factors are identified among the barriers to early recovery.

In the literature, the failure to wean and extubate patients has been reported at a rate of 6.3-45.5%. Bleeding and hemostasis have been reported as potentially modifiable factors [5,9]. Age, female gender, prolonged surgery, and prolonged cross-clamp duration have been shown to be independent risk factors for non-fast-track anesthesia [10]. In our study, 61.1% of the patients were FTCA, and 38.9% were not. Patients who met the FTCA criteria had shorter ICU and hospital stays and lower rates of return to the ICU and mortality. The most common causes of failure to meet the FTCA criteria were cardiovascular complications (55.7%), followed by respiratory complications and surgeon’s refusal both with rates of 15.9%. Some patients met the FTCA requirements but were not allowed to be extubated may reflect the problem of some surgeons continuing old habits. Prolonging the duration of mechanical ventilation and sedation predisposes to many complications [11]. Given the frequent difficulty in modifying cardiac and/or pulmonary risk factors, the factor that was most easily changed or improved in terms of FTCA seems to be the surgeon’s refusal, which affected 15.9% of the patients. Explaining contemporary approaches to those who continue their old habits in this regard will help in FTCA implementation.

Regarding other risk factors for failure to meet the FTCA criteria, ASA and Euro Scores were higher and ejection fraction was lower in the preoperative period. Although it may be recommended to pay attention to the control of comorbidities in the preoperative period, the presence of these diseases is not a changeable factor. Regarding intraoperative and postoperative characteristics, these patients underwent more complex surgical procedures, CPB and operation duration were approximately 30 minutes longer, had longer ICU and hospital stays, and had higher return to ICU and mortality rates. The number of patients who received RBC transfusions was higher in this group.

Several strategies have been proposed in the literature regarding patients who should be prioritized for surgery in times of crisis such as a pandemic. These strategies can be related to the severity and progression of the primary disease and the urgency of a surgical procedure [12,13]. Most recommendations include prioritizing patients with immediate surgical treatment requirements within 72 hours of presentation, such as critical coronary artery disease, need for vasoactive support, a valvular pathology resulting in cardiogenic shock, pulmonary embolism, acute type A aortic dissection, and acute heart failure [14-16]. On the other hand, it is necessary to perform procedures in some way in cases that are not so urgent and are waiting for heart surgery. In the literature published during the COVID-19 pandemic, the use of minimally invasive cardiac procedures in select patients has led to earlier recovery and shorter stay durations [12,17,18]. However, this is far from standard as it requires experienced teams, and not every patient is a candidate.

As a limitation, our single-centered study consisted of a two-month cross-sectional observation. Further long-term studies need to be conducted.

## Conclusions

In our research to reveal the feasibility of and barriers to FTCA, cardiac and respiratory problems were the most common reasons for delayed extubation in the postoperative period. Due to the refusal of the surgical team, it was observed that approximately 16% of the patients remained intubated despite meeting the FTCA requirements. This is the most modifiable obstacle to FTCA in this study. The most important risk factors in cases where FTCA cannot be applied are ASA III score and RBC transfusion; unfortunately, these are not always modifiable. Therefore, strategies to ensure patient turnover in cardiac surgery should aim to optimally control patient comorbidities in the preoperative period, reduce the use of RBC transfusions, and educate surgical and anesthetic staff about FTCA and its benefits as well as help them implement it.
